# Multidisciplinary approach to diagnosis and management of osteosarcoma – a review of the St Vincent's Hospital experience

**DOI:** 10.1186/1477-7800-3-38

**Published:** 2006-11-03

**Authors:** Judith Zhi-Yie Tan, Stephen M Schlicht, Gerard J Powell, David Thomas, John L Slavin, Peter J Smith, Peter FM Choong

**Affiliations:** 1 Department of Medical Imaging, St Vincent's Hospital, Melbourne, Australia; 2 Department of Orthopaedics, St Vincent's Hospital, Melbourne, Australia; 3 Bone and Soft Tissue Sarcoma Service, Peter MacCallum Cancer Centre, Melbourne, Australia; 4 Department of Pathology, St Vincent's Hospital, Melbourne, Australia

## Abstract

**Background:**

Osteosarcoma is the most common primary malignant bone tumour in children and young adults. Despite advances in the diagnosis and management of osteosarcoma, there have been few recent studies describing the experiences of tertiary referral centres. This paper aims to describe and discuss the clinical features, pre-operative work-up, management and outcomes of these patients at St Vincent's Hospital (Melbourne, Australia).

**Methods:**

Retrospective study of fifty-nine consecutive patients managed for osteosarcoma at St Vincent's Hospital between 1995 and 2005.

**Results:**

Median age at diagnosis was 21 (range, 11–84) years. Gender distribution was similar, with thirty-one male and twenty-eight female patients.

Twenty-five patients had osteosarcoma in the femur, eleven each were located in the humerus and tibia, six were identified in the pelvis, and one each in the clavicle, maxilla, fibula, sacrum, ulna and radius.

Pre-operative tissue diagnosis of osteosarcoma was obtained through computed tomography-guided percutaneous biopsy in over ninety percent of patients.

Following initial therapy, over fifty percent of patients remained relapse-free during the follow-up period, with twelve percent and twenty-seven percent of patients documented as having local and distant disease recurrence, respectively. Of patients with recurrent disease, sixty-two percent remained disease-free following subsequent surgical intervention (most commonly, pulmonary metastatectomy).

**Conclusion:**

Patient outcomes can be optimised through a multidisciplinary approach in a tertiary referral centre. At St Vincent's Hospital, survival and relapse rates of patients managed for osteosarcoma compare favourably with the published literature.

## Background

Osteosarcoma is the most common primary bone malignancy in children and young adults [1-5], typically presenting with symptoms of pain or swelling. Older adults may also be affected, usually as a result of sarcomatous transformation of Paget's disease of bone [1-3,6]. Histologically, osteosarcomas are highly aggressive spindle cell neoplasms that produce osteoid. Overall, this malignancy is rare, with an annual incidence of approximately one per 100,000 persons [1-5].

Since the 1970s, when two-year survival rates were fifteen to twenty percent [2], patient outcomes have significantly improved following the introduction of effective multi-agent chemotherapy regimens. In addition, developments in imaging techniques have contributed to more effective management of osteosarcoma. These included computed tomography (CT) and magnetic resonance (MR) evaluation of bone and soft tissue involvement to facilitate surgical planning, use of functional nuclear imaging and CT chest for detection of metastatic disease, and imaging-guided percutaneous biopsy to enable histopathological diagnosis with minimal morbidity and complication risks. Furthermore, advances in limb-salvage surgery and reconstructive techniques in selected patients have enabled preservation of limb function without compromising oncological outcomes [2,5].

A retrospective review of the experience at St Vincent's Hospital with osteosarcoma was performed in order to evaluate the diagnosis and management of these patients, and the outcomes achieved.

## Materials and methods

Between 1995 and 2005, fifty-nine consecutive patients were diagnosed and managed for osteosarcoma at St Vincent's Hospital, Melbourne, Australia. A retrospective review of these patients was undertaken, with data on age at diagnosis, gender, symptoms, site of the tumour, treatment regimen, follow-up and outcomes, compiled from the clinical notes, outpatient correspondences and histopathology reports.

## Results

### Age and gender

Median age at diagnosis was 21 (range, 11–84) years.

There were thirty-one male and twenty-eight female patients.

### Symptoms (see Figure 1)

**Figure 1:**
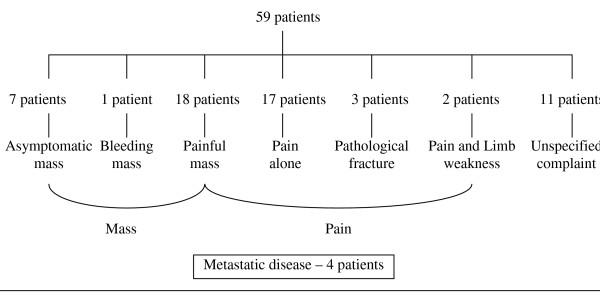
Presenting symptoms.

Pain was the most common presenting symptom, being the sole symptom in seventeen patients. Seven patients presented with an asymptomatic mass lesion, eighteen patients complained of a painful mass lesion, three patients presented with a pathological fracture (two associated with pain prior to diagnosis of fracture), two patients experienced pain and limb weakness, and one patient presented with bleeding. Four patients were found to have metastatic disease at diagnosis. In the remaining eleven patients, the presenting complaint was not specified.

### Site of tumour (see Figure 2)

**Figure 2:**
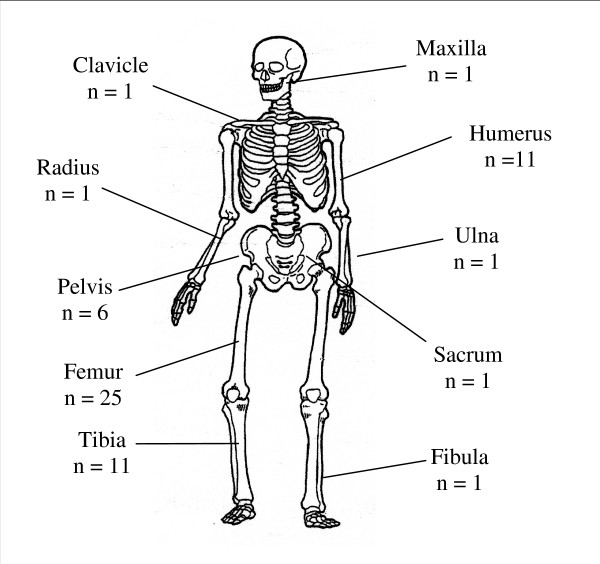
Frequency and Location of Osteosarcoma.

Twenty-five osteosarcomas were located in the femur; of these, nineteen were in the distal femur, one in the proximal femur and in five the location was unspecified. Of the other sites of tumour, eleven each were identified in the humerus and tibia, and six in the pelvis. One case of osteosarcoma was diagnosed in each of the following locations: clavicle, maxilla, fibula, sacrum, ulna and radius.

### Pre-operative diagnosis, treatment and outcome

Diagnosis and pre-operative assessment was performed using a combination of plain radiographs, bone scan, computed tomography scan (including of the chest), magnetic resonance imaging and functional nuclear medicine including thallium scintigraphy and positron emission tomography (PET)-CT scan. In addition, imaging-guided CT biopsy was performed in fifty-one patients; of these, twenty-six were performed at St Vincent's Hospital and twenty-five at external institutions. In five of these patients, one of whom was initially biopsied outside St Vincent's Hospital, open biopsy was required for definitive diagnosis of osteosarcoma. In eight patients, presumptive diagnosis of osteosarcoma was made pre-operatively and confirmed by histopathology following surgical resection.

Fifty-five patients were managed with surgical resection, including thirty patients who underwent limb-salvage surgery with reconstruction. All but five patients received chemotherapy; of patients who were administered chemotherapy, thirty-four patients received both neo-adjuvant and adjuvant treatment, eighteen patients had only neo-adjuvant and two patients had only adjuvant chemotherapy. Three patients received radiotherapy, one as palliative management.

**Figure 3:**
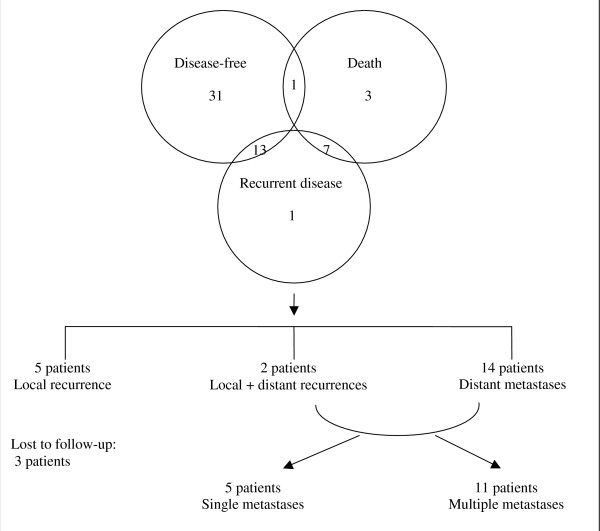
Patient Outcomes.

Median duration of follow-up, defined as the time from diagnosis to that of the most recent clinical evaluation, was twenty-eight (range, 1–111) months. Forty-five patients were disease-free at the most recent follow-up (see Figure 3). During the follow-up period, eleven patients had died; of these, only one death was unrelated to osteosarcoma. Twenty-one patients were found to have had recurrent disease – in five patients there was local recurrence, fourteen patients had metastatic disease and two patients had both local recurrence and metastatic disease. Of the patients with known sites of metastatic disease, five had single metastases while eleven had multiple metastases.

## Discussion

Osteosarcoma is the most common primary bone malignancy in children and adolescents, with an overall incidence of approximately one per 100,000 per year [1-5]. Whilst most osteosarcomas are de novo tumours of unknown aetiology, secondary causes include ionising radiation, hereditary retinoblastoma, Paget's disease of bone, enchondromatosis, hereditary multiple exostoses and fibrous dysplasia [1-3,6].

Osteosarcoma typically presents between five and thirty years of age [5], although a bimodal age distribution has been noted, with a first peak at ten to twenty years of age and a second peak in older adults aged in the fifth and sixth decades of life [2]. The age of the patients in our study were aged between eleven and eighty-four years, of whom sixty-eight percent (n = 40) at diagnosis were aged ten to thirty years inclusive.

As previously reported, there was no significant gender preponderance in patients affected by osteosarcoma in this study [1,3], although the incidence has been described as being more frequent in males (1.5:1) [2,7].

Patients typically present with pain or swelling in a bone or joint, and occasionally a pathologic fracture secondary to marked weakening of bone at the site of the lesion, which may occur on the background of minor trauma or vigorous physical exercise [1,4-6]. Symptoms may be present for several months (on average, three to four months, but often exceeding six months) before a diagnosis is made [2-4,8]. Most patients present with localised disease – eighty percent of these tumours are non-metastatic at time of presentation [2,3,5]. In this series, only seven percent of patients presented with metastatic disease. Osteosarcoma most commonly metastasises to lung (ninety percent) and bone (ten percent) [1,7]. In patients treated with surgical resection and/or radiotherapy without chemotherapy, approximately eighty percent with localised disease at presentation developed secondary disease related to micrometastases [2].

Osteosarcomas can occur in any bone but most commonly affects the metaphysis of long bones in the appendicular skeleton (eighty percent) [7]. The most common sites affected are: distal femur (thirty-five percent), proximal tibia (twenty percent), and proximal humerus (ten percent) [1-3,5,7]; consistent with previous studies, over half (sixty-one percent, n = 36) of osteosarcomas diagnosed in this study originated from the knee area [1,7]. Osteosarcoma can also affect the axial skeleton, although this occurs in less than ten percent of the paediatric age group; in these patients, the pelvis is most common site [2]. Ten percent of osteosarcomas present in the head and neck region, of which the average age of presentation is between the third and fourth decades [4]; only one patient in this series had maxillary osteosarcoma.

**Figure 4:**
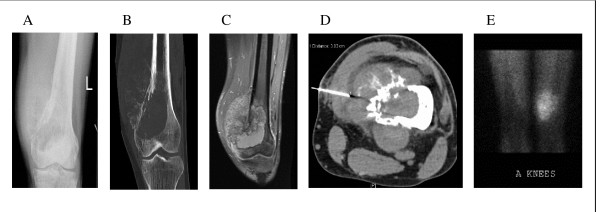
Imaging features of osteosarcoma. Panel A is a plain radiograph demonstrating a lytic destructive lesion with malignant new bone formation involving the medial aspect of left distal femur. Panel B is a coronal CT image showing typical periosteal new bone formation with cortical destruction associated with a lytic bony lesion involving the left distal femur. Panel C is an enhanced MR image demonstrating an eccentric left distal femoral metaphyseal mass with a large extra-osseous component. Panel D is an axial image of a CT-guided biopsy sampling of the extra-osseous mass. Panel E is an image from a thallium bone study showing uptake overlying the distal left femur indicative of high grade tumour at this site.

The diagnosis of osteosarcoma is based on the demonstration of typical radiographic findings (see Figure 4) in association with characteristic cytologic features.

### 1. Plain radiographs

On plain radiographs, osteosarcomas typically appear as an ill-defined mixed sclerotic and lytic lesion arising in the metaphyseal region of the involved bone, with destruction of the bony cortex, formation of a soft tissue mass, and ossification [2,5,7]. Periosteal new bone formation with elevation of the cortex may also be present, forming the appearance of a Codman triangle [2,3,7].

### 2. CT

CT scanning of the affected bone is superior to MR imaging for the assessment of new bone formation and cortical changes. In addition, CT chest is mandatory for detection of pulmonary metastases, the presence of which affect management and prognosis.

### 3. MR imaging

MR imaging, with T1- and T2-weighted and fat-suppressed sequences, is superior to CT in assessing for skip lesions (on longitudinal images) and the extent of bone and soft tissue involvement (on axial images). In particular, the intra-osseous extent of tumour and its proximity to muscle groups, subcutaneous fat, joints and neurovascular structures [2,3,5] for surgical planning is determined with the most specificity on T1-weighted sequences but with the most sensitivity on short tau inversion recovery sequences [8]. Dense sclerosis within neoplastic bone gives a hypointense signal on all sequences, especially on T2-weighted images, while tumour within the marrow appears hypointense on T1-weighted images and hyperintense on T2-weighted images [8]. MR axial T1-weighted fat-suppressed post-contrast images may be used to assess tumour viability and response to neo-adjuvant chemotherapy prior to surgical resection [8].

### 4. Nuclear medicine

Functional nuclear imaging assesses the metabolic activity of tumour.

Triple-phase whole-body scintigraphy is useful for detection of bone metastases. In instances where MR imaging is unhelpful for monitoring tumour response to neo-adjuvant chemotherapy and detection of local recurrence, thallium scintigraphy may be performed.

PET-CT is emerging as a tool for non-invasive assessment of neo-adjuvant chemotherapy response in osteosarcoma [9-12], and may potentially have a role in post-treatment follow-up detection of metastatic disease, although it has been reported in small studies as being less sensitive than bone scintigraphy and thoracic CT for detection of osseous and pulmonary metastases, respectively [13,14]. Lack of access to this imaging modality also limits its current use.

### 5. CT-guided biopsy

Although radiographic appearance and location of tumour is predictive of osteosarcoma in two-thirds of cases, pathologic confirmation by biopsy is more definitive. Percutaneous biopsy is minimally invasive, requires minimal wound healing, and is associated with a lower risk of infection, contamination and post-biopsy fracture [5]. In particular, core biopsies performed under local anaesthaesia are capable of obtaining an adequate specimen with tissue architecture maintained, enabling diagnostic accuracy of over ninety-five percent [2]. In this study, ninety percent (n = 46) of patients percutaneously biopsied under imaging (CT) guidance had histologic confirmation of osteosarcoma pre-operatively, with only four CT-guided core biopsies performed at this institution requiring an open biopsy for definitive diagnosis of osteosarcoma. Improperly performed biopsies, more common in non-tertiary institutions, are associated with increased errors in diagnosis, obtaining non-representative tissue, and adverse outcomes including amputation and local recurrence [6].

Histologically, osteosarcoma is a malignant spindle-cell neoplasm that produces osteoid [7]. High-grade osteosarcoma can be classified into four histologic subtypes (osteoblastic, fibroblastic, chondroblastic and telangiectatic) based on the predominant type of matrix within the tumour. These subtypes appear to have similar prognoses, thus rendering the value of these distinctions unclear. However, tumour grade and identification of two rare clinical subtypes (parosteal and periosteal osteosarcomas) appear to be more significant as prognostic indicators, with these subtypes being associated with a lower risk of metastasis and more favourable outcome with surgical resection alone [1,6].

The most widely used staging system is the Enneking system, which categorises localised malignant bone tumours by grade (low and high grades being Stages 1 and 2, respectively) and tumour extension through the cortex (intra-compartmental and extra-compartmental being classified as subtypes A and B, respectively); patients with metastatic disease are classified as having Stage 3 disease. Typically in younger patients, high-grade osteosarcomas extend through the cortex early in their natural history – thus, most patients are Stage 2B or 3 at presentation [2].

The differential diagnoses include osteomyelitis, osteosarcoma and other primary bone tumours (myositis ossificans, aneurysmal bone cyst, Ewing's sarcoma, chondrosarcoma, giant cell tumour, osteoblastoma, fibrous dysplasia), and, although infrequent in the paediatric population, metastatic bone disease (lymphoma, neuroblastoma, rhabdomyosarcoma). The location of the tumour within the bone and the skeletal location assist in distinguishing osteosarcoma from Ewing's sarcoma, the second most frequent type of bone tumour in children and adolescents.

The management of osteosarcoma is based primarily on neo-adjuvant and adjuvant chemotherapy and surgical resection; radiotherapy is not effective as osteosarcomas are relatively radioresistant. Since 1970, when osteosarcoma was treated with amputation and/or radiotherapy and more than eighty percent of patients developed metastatic disease following therapy [3,5], advances in chemotherapeutic regimens, surgical techniques and radiologic staging studies have enabled ninety to ninety-five percent of patients to be treated with limb-sparing resection and reconstruction. Survival rates of up to sixty percent at five years [3] and relapse-free rates of sixty to eighty percent [5] in patients with localised disease at presentation have been described.

The most consistent prognostic factor at diagnosis is the presence of clinically detectable metastatic disease [8]. Patients presenting with localised disease have the best outcome, with a five-year relapse-free survival rate of sixty to seventy percent [1]. The prognosis of patients with metastatic disease at diagnosis is poor and appears to be related to the number and resectability of pulmonary nodules, and response to preoperative therapy [1-3]; in one series the survival rate was only eleven percent despite intensive chemotherapy [3].

The site of the primary tumour is also of prognostic value, with axial lesions conferring a poorer outcome, especially if located in the pelvis [1,2].

The histologic response to neo-adjuvant chemotherapy is also a consistent prognostic factor, demonstrated either by magnetic resonance imaging or nuclear scintigraphy prior to resection, or by histologic analysis of the resection specimen [2,3]. In patients with a good histologic response, seventy-five percent are relapse-free three years following therapy [2].

Although patients with recurrent disease generally have a poor prognosis, this is dependent on the type of therapy given previously, the duration of remission, and the extent of metastases [2,3]. With aggressive treatment, the five-year survival rate in patients with pulmonary metastatic recurrence may reach forty percent [2], and in patients with recurrent disease confined to one or a limited number of pulmonary nodules more than one year after initial chemotherapy, cure after complete resection of all nodules is approximately twenty-five percent [1].

The St Vincent's Hospital experience with management of osteosarcoma compares favourably with published literature, with over half of our patients (n = 31) remaining relapse-free during the follow-up period following initial surgical resection and chemotherapy. Only twelve percent (n = 7) of patients had localised disease relapse and twenty-seven percent (n = 16) of patients following initial curative management subsequently developed distant disease, the majority (eighty percent) being pulmonary metastases. Significantly, sixty-two percent (n = 13) of patients with recurrent disease remained disease-free following subsequent surgical resection(s) within the follow-up period.

## Conclusion

The St Vincent's experience has been comparable to the published literature in terms of the patient demographics (age at diagnosis, gender distribution), clinical features (presenting symptoms, site of tumour) and outcomes (survival and relapse rates). Through a multidisciplinary approach in a tertiary referral centre, patient outcomes may be optimised. Use of multiple imaging modalities, including CT-guided biopsy, to demonstrate typical radiographic and histopathologic features of osteosarcoma facilitates surgical planning and appropriate use of neo-adjuvant chemotherapy. Follow-up is essential following initial therapy, as patients may relapse with metastatic disease, most commonly to the lungs, which may be treatable with surgery and chemotherapy.

## Abbreviations

CT computed tomography

MR magnetic resonance

PET positron emission tomography

## Competing interests

The author(s) declare that they have no competing interests.

## Authors' contributions

J. Z-Y Tan participated in the study design, collated the data, performed the statistical analysis and drafted the manuscript.

S. M. Schlicht conceived the study, participated in the study design, helped draft the manuscript, carried out the percutaneous bone biopsies and reviewed the imaging studies.

G. J. Powell and P. F. M. Choong performed the surgical procedures and obtained pathology samples.

D. Thomas provided medical oncology expertise regarding neo-adjuvant and adjuvant chemotherapy regimens.

J. L. Slavin reviewed the histopathology.

P. J. Smith reviewed the imaging studies.

All authors read and approved the final manuscript.
